# Understanding factors that influence physical activity behavior in people with developmental coordination disorder (DCD): a mixed-methods convergent integrated systematic review

**DOI:** 10.3389/fnhum.2023.1274510

**Published:** 2023-12-13

**Authors:** Catherine Purcell, Nadja Schott, Victoria Rapos, Jill G. Zwicker, Kate Wilmut

**Affiliations:** ^1^School of Healthcare Sciences, Cardiff University, Cardiff, United Kingdom; ^2^Institute of Sport and Movement Science, University of Stuttgart, Stuttgart, Germany; ^3^Rehabilitation Sciences, The University of British Columbia, Vancouver, BC, Canada; ^4^Brain, Behavior, and Development, British Columbia Children’s Hospital Research Institute, Vancouver, BC, Canada; ^5^Department of Occupational Science and Occupational Therapy, The University of British Columbia, Vancouver, BC, Canada; ^6^Centre for Psychological Research, Oxford Brookes University, Oxford, United Kingdom

**Keywords:** developmental coordination disorder, motor skills disorder, physical activity, COM-B, behavior change

## Abstract

This systematic review synthesizes the literature on physical activity amongst people with DCD using the COM-B framework. The review questions were: (1) what is the Capability (C), Opportunity (O) and Motivation (M) for physical activity and (2) what does physical activity behavior (B) look like? A mixed-methods systematic review was conducted by searching eight databases (PubMed, APA PsycINFO, EMBASE, Scopus, Child Development and Adolescent Studies, Cochrane Library, Web of Science, CINAHL) up to July 2023. Data were extracted, thematically analyzed, and mapped to the COM-B model. The quality of studies was assessed with the Joanna Briggs Institute (JBI) critical appraisal tool. The protocol was registered with PROSPERO (CRD42022319127). Forty-three papers, 42 of which related to children, were included. Fifteen aligned with physical activity behavior, nine with physical capability, thirteen with psychological capability, one with social opportunity, one with physical opportunity, one with reflective motivation and three with automatic motivation. Pre-school-aged children with DCD engage in comparable levels of physical activity behavior, but differences emerge from 6 years of age. Characteristics of DCD result in reduced physical capability and less varied participation in physical activity. This impacts psychological capability, whereby lower self-perceptions result in a negative feedback loop and reduce the motivation to participate. Barriers relating to social opportunities may result in poor reflective and automatic motivation, although there is evidence that interventions can enhance enjoyment in the short term.

## 1 Introduction

Physical inactivity is one of the leading risk factors for premature mortality worldwide (World Health Organization [WHO], 2022), accounting for 5.3 million deaths annually (Lee et al., 2012). In the UK alone, physical inactivity contributes to one in six deaths and is estimated to cost £7.4 billion annually (Public Health England, 2016). Physical inactivity increases the societal and economic burden of mental and physical ill-health; however, despite the clear evidence of the health benefits of being physically active, over a quarter of the world’s adult population is insufficiently physically active, and 81% of 11–17 year-olds were insufficiently physically active in 2022 (World Health Organization [WHO], 2022). Promoting physical activity is, therefore, a global public health priority (World Health Organization [WHO], 2022).

However, some families experience significant inequalities in opportunities for physical activity, such as families of children living with disabilities, who often experience greater environmental barriers (World Health Organization [WHO], 2020). One such group is children and adults with developmental coordination disorder (DCD). DCD is a neurodevelopmental disorder affecting 5–15% of school-aged children (Hamilton, 2002) that significantly impacts a child’s ability to learn motor skills and perform everyday activities, including getting dressed, tying shoelaces, using cutlery, handwriting, playing games or sports, or riding a bicycle (Zwicker et al., 2012). Ultimately, these motor deficits harm academic performance, vocational choices and leisure pursuits (Zwicker et al., 2012). Secondary consequences of DCD include low self-esteem, depression, anxiety, loneliness, problems with peers and withdrawal from participating in physical and social activities (Zwicker et al., 2018; Izadi-Najafabadi et al., 2019; Harris et al., 2021). Furthermore, the motor and psychosocial difficulties associated with DCD profoundly impact quality of life (Zwicker et al., 2018) and persist in adulthood (Harris et al., 2021).

Previous research has explored physical inactivity in children and adults with DCD and identified that people with DCD are less physically fit (Schott et al., 2007), less physically active (Cairney et al., 2012), have lower perceived athletic competence and tend to avoid participation in physical activity (Rivilis et al., 2011). These findings are supported by a recent systematic review that explored differences in physical activity levels and the impact of these differences (Mercê et al., 2023). The authors concluded that the 16 included studies identified lower levels of moderate and vigorous physical activity amongst children with DCD, with implications across physical and psychological domains reported (Mercê et al., 2023). A recent scoping review also examined psychosocial factors related to physical activity among children with DCD based on social cognitive theory, self-determination theory and the theory of planned behavior (Kwan et al., 2022). The authors concluded from the 14 papers that physical literacy-based interventions targeting perceived motor competence and motivation might effectively promote physical activity in children with DCD (Kwan et al., 2022).

However, despite these findings and increased intervention efforts to increase physical activity amongst people with DCD, most lack evidence-based behavior change theories. Behavior science approaches are based on the idea that successful behavior change depends first and foremost on a clear definition of the problem: who needs to change what behavior, in what way and what is required to do so? Once the behavior is clearly defined, in the present case that people with DCD avoid physical activity or engage in physical activity only to a limited extent, behavior change interventions can be developed.

Many behavior science models assume that successful behavior change interventions must consider three essential aspects: motivation, competence and situation. The COM-B model (Michie et al., 2011) was developed following a comprehensive review and consultation of 19 existing frameworks of behavior change interventions, none of which incorporated a full range of intervention functions or policies, therefore the COM-B provides a comprehensive and coherent link to a model of behavior. The COM-B model (Michie et al., 2011) posits that Behavior (B) occurs as a result of a bi-directional interaction between three components: Capability (C), Opportunity (O) and Motivation (M) and, as such, can contribute insights into physical activity behavior. This model explains that to perform a particular behavior; one must feel they are physically and psychologically able to do so (C), have the physical and social opportunity (O) and want or need to carry out the behavior more than other competing behaviors (M).

While some of the previous literature and systematic reviews have explored individual components of the COM-B, this is the first systematic review that brings the COM-B components together to better understand the physical activity behavior of people with DCD. This is necessary to develop future behavior change interventions, using the Behavior Change Wheel, that aim to increase physical activity. Without a critical overview of the literature relating to capability, opportunity, and motivation for physical activity amongst people with DCD, there is a risk that interventions focus on components that do not result in behavior change. Additionally, a comprehensive overview of the literature enables future research to focus on any gaps identified, strengthening the evidence for future interventions. Therefore, this systematic review addressed the following questions: what is this group’s capability, opportunity and motivation for physical activity? and (Lee et al., 2012) what does physical activity behavior look like amongst people with DCD?

## 2 Methods

This systematic review was informed by the Joanna Briggs Institute (JBI) methodology for conducting mixed-method systematic reviews (Stern et al., 2020). The review protocol was registered on PROSPERO in March 2022 (reference number: CRD42022319127).

### 2.1 Eligibility criteria

We developed comprehensive inclusion and exclusion criteria to judge the eligibility of potential publications involving people with DCD and physical activity outcomes for inclusion in this systematic review. The criteria were developed *a priori* based on the results of a preliminary scoping search in CINAHL and were piloted on two papers identified through the initial search. CP and one other reviewer, KW, independently applied the eligibility criteria. The reviewers discussed potential changes; however, the eligibility criteria did not need to be updated prior to application. The full eligibility criteria are detailed below.

#### 2.1.1 Study design

Qualitative, quantitative, and mixed-method studies written in any language and peer-reviewed were included. This review did not include systematic reviews, meta-analyses, study and review protocols, commentaries, editorials, gray literature, conference posters or abstracts, although reference lists were used to enhance search results. Only English language articles were identified, so translation was unnecessary.

#### 2.1.2 Participants

We included studies concerning children (under 18 years) or adults (18+ years) who met the Diagnostic Statistical Manual of Mental Disorders (DSM) or International Classification of Diseases (ICD) criteria for DCD (or at least 2 out of 4 criteria). Studies that reported co-occurring diagnoses/characteristics were included if the article’s primary focus was DCD. Articles were excluded if they did not include a standardized motor assessment, or where another condition or visual impairment could explain the motor difficulties, or where motor difficulties were a consequence of a lack of opportunity.

#### 2.1.3 Intervention

The focus of this systematic review was not on interventions; however, any studies that included interventions, even when the primary outcomes were not relevant to this review, were included if relevant baseline data were reported.

#### 2.1.4 Comparators

For interventions including randomized controlled trials or pre-post intervention studies of any duration, articles were included if a comparator group did not receive a physical activity intervention or if the comparator group was a typically developing (TD) group.

#### 2.1.5 Outcomes

Articles that reported outcomes in line with physical activity Behavior (B), Capability (C), Opportunity (O), and Motivation (M) were included. In addition, we considered other outcomes if they were measured alongside a COM-B component. A non-exhaustive list of examples of eligible outcomes are presented in Table 1.

**TABLE 1:** Examples of facilitators to physical activity behavior and common measures framed within the COM-B.

COM-B components	Possible facilitators of physical activity behavior	Possible measures
***Capability: individual’s physical and psychological capacity to engage in the behavior***		
*Psychological: the capacity to engage in necessary thought processes*	Need knowledge of suitable local activity opportunities Need to know about easy and manageable activities that are safe	Children’s self-perception and adequacy in predilection for physical activity
*Physical: the capacity to engage in the necessary physical processes*	Motor coordination problems Reduced fitness	Adolescent Motor Competence Questionnaire, Developmental Coordination Disorder Questionnaire, Movement Assessment Battery for Children/Movement Assessment Battery for Children – 2nd ed, Test of Gross Motor Development, Bruininks Oseretsky Test of Motor Proficiency/Bruininks Oseretsky Test – 2nd ed, Canadian Agility and Movement Skill Assessment; Physical Activity Questionnaire
***Opportunity: all factors lying outside the individual that makes the performance of the behavior possible or prompt it***		
*Social: cultural milieu that dictates the way we think about things*	Family and peers provide encouragement to be active. Want to be active with other people with DCD who understand their current situations. Opportunity to be part of a group, to create accountability and provide encouragement.	Social support for exercise behavior scale
*Physical: physical opportunity provided by the environment*	Need accessible and pleasant walking routes or groups —good pavements or footpaths, safe and greenspace. Need appropriate and accessible recreational spaces and differentiated programs	Neighborhood environment scale Presence of recreational facilities index Participation and environment measure for children and youth
***Motivation: all brain processes that energize and direct behavior***		
*Automatic: emotions and impulses arising from associative learning and/or innate dispositions*	Social interaction with other people is a motivation to be active. Want to take part in physical activity that is fun and enjoyable	Physical literacy in children questionnaire Physical exercise self-efficacy scale Exercise self-identity scale Perceived behavioral control. Positive and negative affect schedule short form Children’s assessment of participation and enjoyment/preferences for activities of children
*Reflective: evaluations and plans*	Understand the physical and mental health benefits of physical activity	Canadian assessment of physical literacy Physical literacy in children questionnaire

### 2.2 Search strategy

We conducted a preliminary search of CINAHL on the 16th of March 2022 to scope the literature relevant to the review questions. The scoping exercise helped ensure that there were no current or ongoing reviews on the topic of interest, refine the aims and eligibility criteria for this systematic review, estimate the amount of published work available, and, therefore, the resources needed to complete this systematic review. Relevant articles identified from the scoping search of CINAHL were also used to develop a full search strategy; keywords in the titles and abstracts and the index terms used to describe the articles were organized into search strings. The search period was not limited. We used the following keywords and MeSH (medical subject heading) terms: developmental coordination disorder; motor skills disorders; DCD; probable DCD; significant motor difficulties; motor development; dyspraxia; motor competence; physical activity; sedentary behavior; exercise; physical performance; sport; aerobic exercise; fitness; motor activities; anaerobic exercise and participation.

### 2.3 Data sources

We searched the following electronic databases for peer-reviewed articles between the 6th May 2022 and the 27th May 2022: PubMed; APA PsycINFO; EMBASE; Scopus; Child Development and Adolescent Studies; Cochrane Library; Web of Science; CINAHL via EBSCO and ERIC. A final search was conducted on the 10th July 2023 to ensure any articles published after the 27th May were captured; no additional articles were identified for inclusion. No date restrictions were applied to the searches; all publications up to the date of the searches were considered.

### 2.4 Article screening

**FIGURE 1:**
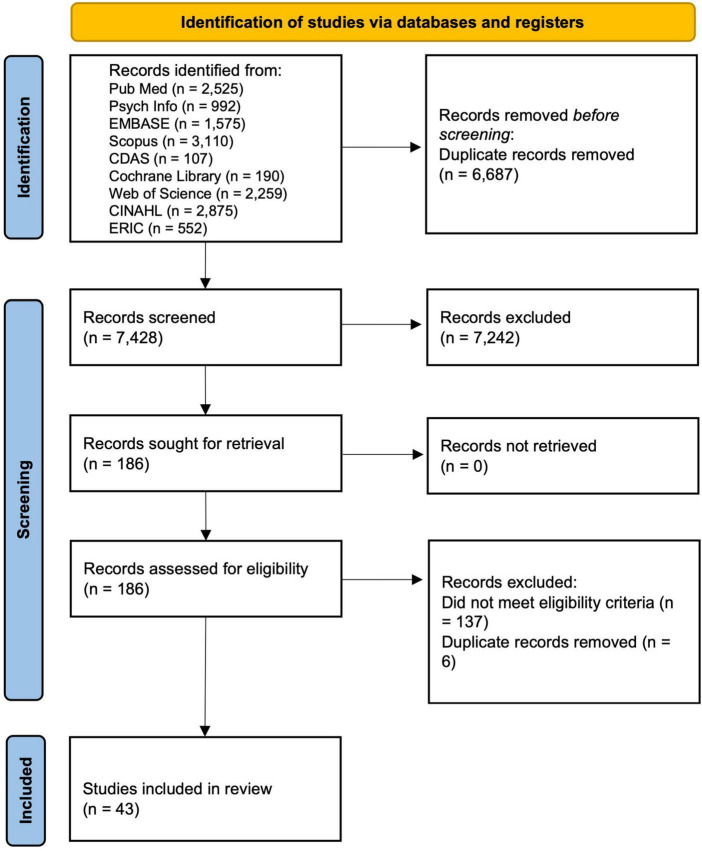
PRISMA flow chart.

References were imported into Rayyan (Ouzzani et al., 2016), and duplicates were removed. Initially, the titles and abstracts of articles were screened independently by CP and KW against the eligibility criteria, and any conflicts that arose were resolved through discussion and, where necessary, by the third reviewer, NS. The screening process was reported in the Preferred Reporting Items for Systematic Reviews and Meta-analysis (PRISMA) 2020 flow diagram (Page et al., 2021) (see Figure 1).

### 2.5 Critical appraisal

We assessed the methodological quality of included articles using the established JBI critical appraisal tools for the following study designs: Randomized Controlled Trials (RCTs) and quasi-experimental studies (Tufanaru et al., 2017); analytical cross-sectional studies, case reports and cohort studies (Moola et al., 2015) and qualitative research (Lockwood et al., 2015).

We adopted the method and classification outlined by Edwards et al. (2016) to judge quality, and included articles were assessed against the pre-determined criteria. Quantitative and qualitative components of mixed-method studies were appraised separately using the appropriate critical appraisal instruments. Each paper received an overall score based on the number of criteria met (13 for RCTs, 10 for qualitative and cohort studies, 9 for quasi-experimental studies and 8 for analytical cross-sectional studies and case reports). A point was deducted from the total available score if a criterion was considered not applicable to a particular article. A percentage score allowed the normalization of scores; 0–40% were considered low quality, 40–70% moderate quality and 70–100% high quality.

Each article was assessed independently by one of the authors, and then all scores were checked by a paired reviewer (e.g., CP reviewed KW).

### 2.6 Data extraction

A piloted template (Supplementary material, online supporting information) was used to extract the study design, sample characteristics, diagnostic criteria, methodology and summary outcomes. Data were independently extracted by a single author and checked by a paired reviewer. The review team conducted consensus checks and resolved discrepancies through discussion. Studies were grouped into one of seven categories: physical capability; psychological capability (C); physical opportunity; social opportunity (O); reflective motivation; automatic motivation (M) and behavior (B).

### 2.7 Data transformation

One reviewer (CP) employed a convergent integrated approach to synthesize the data: this involved narrative interpretation of the quantitative results from experimental studies (including the quantitative component of mixed-method studies) in a way that answered the review questions by a repeated detailed examination. Specifically, quantitative and qualitative findings were initially synthesized separately; quantitative data was converted into “qualitized data” through transformation into narrative interpretation, followed by integration of both sets of findings. This approach provides greater insights and preserves the integrity of both sets of findings (Stern et al., 2020).

### 2.8 Data synthesis and integration

We narratively integrated quantitative and qualitative data at the interpretation level using the COM-B components to identify the relationship between and within quantitative and qualitative findings. The narrative summary was scrutinized by the review team for accuracy.

## 3 Results

### 3.1 Characteristics of included studies

We screened 7,428 titles and abstracts and assessed 186 full texts for eligibility. Forty-three papers met the eligibility criteria and were included in the review (see Figure 1).

The characteristics of studies included in this systematic review are presented in Table 2. All included studies considered children or adolescents, apart from one (Tan et al., 2022), which followed up with participants at the age of 25 years.

**TABLE 2:** Characteristics of the included studies, including author [reference], study design, percentage quality score, sample, diagnostic criteria and summary of outcome.

Author	Study design	% Quality score	Sample	DMS IV and DSM 5*		DSM IV		DSM 5		Summary outcome
				Criterion A	Criterion B	Criterion C	Criterion D	Criterion C	Criterion D	
				Poor acquisition and execution of motor skills	The deficits interfere with activities of daily living	Not due to general medical condition and does not meet criteria for PDD	Motor difficulties are in excess of any intellectual deficits	Onset of symptoms is in the early developmental period	Not better explained by intellectual, visual or neurological condition	
**Behavior**										
Cairney et al., 2006	Long	100%	44 DCD 537 TD 9–14 years	BOTMP-SF		Children with known learning disabilities and physical health problems were excluded				Children with DCD participated less in structured and free play activities, but this did not change with age.
Poulsen et al., 2008 **	CS	75%	60 boys with DCD 113 boys without DCD 10–13 years	MABC-2 <15th	Parent report	Excluded if had a diagnosed neurological/psychiatric condition	Excluded if IQ < 70 on SIT-R3			LPA and MVPA both sig lower in DCD vs peers. Boys with DCD spent less time in structured and unstructured PA compared to peers.
Cairney et al., 2010 Same sample as PHAST study	QE	75%	111 pDCD 1972 TD Start age for sample: 9 years 11 months	BOTMP and MABC <6th			Kaufman brief IQ test			Divergence in free-play activity occurs for females with probable DCD, but not for males.
Green et al., 2011	QE	75%	193 pDCD 4138 TD 7 years and 12 years	ALSAPC coordination test (one item per scale from the MABC), <15th	23 item ADL scale completed by parent	Excluded those with known visual/neurological condition	Excluded those scoring below 70 on short form of WISC-III			Boys with p-DCD were less physically active than boys without DCD (no group difference in girls).
Baerg et al., 2011 Same sample as PHAST study	CS	88%	32 DCD (12.8 years) 30 DCD/ADHD (12.9 years) 48 TD (12.7 years)	MABC-2, <15th			Used the KBIT-2 to determine typical IQ			Girls: DCD/ADHD > control for daily step count. No other differences in step count. Boys: no differences in daily step count between groups.
Poulsen et al., 2011a	CS	75%	60 DCD boys 113 boys without DCD 10–12 years 11 months	MABC, <15th	Parent identified difficulties with daily living skills	Excluded if had a previously diagnosed neurological or medical disorder	Excluded if IQ < 70 on SIT-R3			Boys with DCD reported fewer (MVPA) activities, and majority of PA in DCD group were completed individually or in the home environment.
Beutum et al., 2013	CS	75%	9 pDCD (8 years, 10 months) 9 TD (8 years, 11 months)	MABC-2, <15th	Parents reported on activities of daily living		Teachers reported no cognitive difficulties			Children with DCD: participated in less MVPA, had higher BMIs, decreased strength and cardiovascular fitness.
Cermak et al., 2015 **	CS	75%	US: 31 DCD (9.3 years) and 44 TD (9.5 years) Israel: 22 DCD (8.7 years) and 21 TD (girls [9.0 years])	MABC-2, <15th	Parent’s report challenges in ADL				Parental report regarding medical history	In both Israel and the US, children with DCD demonstrated reduced physical activity, increased sedentary behavior, poorer fitness and increased overweight vs typical children.
Howie et al., 2016	RCT	62%	21 DCD or rDCD 10–12 years	MABC-2	DCD-Q				Parents reported no known behavioral or neurological disorders	No significant differences in time spent in sedentary, light, moderate or vigorous physical activity between the intervention and control periods.
Howie et al., 2017	RCT	62%	Sample as Howie et al. (2016)					Sample as Howie et al. (2016)		Participants described being more confident, stronger, having improved fitness and an increased willingness to participate in sports and physical activity.
King-Dowling et al., 2019 Same sample as CATCH study	CS	75%	111 pDCD (4.9 years) 177 rDCD (4.9 years) 301 TD (5 years)	MABC-2, <5th for pDCD and 6th–16th for rDCD	Determined via interview with parents			Young children, so in early developmental period	Excluded if had a diagnosis of a medical condition affecting motor control	No group differences for amounts of daily activity. Children with p-DCD accumulate MVPA in shorter bouts.
Cairney et al., 2019 Same sample as CATCH study	CS	100%	287 rDCD 301 TD 4–5 years of age	MABC-2, <16th	Determined via parent semi-structured interview				Excluded those with any known neurological or physical condition which might affect motor control	No group differences for BMI percentile or physical activity. Children in the rDCD group had significantly lower aerobic and musculoskeletal fitness, and larger waist circumference.
James et al., 2021 Same sample as CATACH study	CS	75%	288 at rDCD (4.9 years) 301 TD (5.0 years)	MABC-2, <16th				Young children, so in early developmental period	Parents reported medical history	When adjusting symptoms of ADHD, children at risk of DCD are less active than their TD peers.
Brown et al., 2021 Same sample as CATCH study	CS	75%	288 rDCD (4.87 years) 301 TD (5 years)	MABC-2, <16th				Young children, so in early developmental period	Excluded those with a physical disability, or medical condition which might affect motor control	Movement compositions were relatively similar for TD preschool-age children and those classified as at risk of DCD.
Tan et al., 2022	Long	71%	30 DCD 53 rDCD 575 TD 5 years and 25 years	Zurich Neuromotor assessment	Determined via clinical interviews with parents					Participants at risk of DCD had a lower total number of steps than those not at risk. Modeling indicated that DCD risk status increased time spent in sedentary light activity and decreased time spent in MVPA.
**Physical capability**										
Fong et al., 2011	CS	100%	81 DCD (∼8 years) 67 TD (∼8 years)	BOMTP, composite score < 42	Reported difficulties with ADL	Pediatrician ruled out neurological conditions which might explain the difficulties	No intellectual impairment			Children with DCD participated in fewer activities and participated less often than peers. Companionship, location of participation, and enjoyment level did not differ between the two groups.
Joshi et al., 2015 Same sample as PHAST study	Long	100%	103 pDCD 2175 TD 9–10 years	Bruininks–Oseretsky Test of Motor Proficiency 1st Edition (BOT-SF)					Excluded children with known physical or learning difficulties	Higher BMI and waist circumference found in DCD compared to peers. This difference increased over time. Physical activity did not mediate or moderate the relationship between DCD and body composition.
Yu et al., 2016a	RCT	69%	38 with DCD (DCD[Exp] = 22; DCD[Con] = 16) 46 TD (TD[Exp] = 17; TD[Con] = 29) Aged 9–10 years	MABC-2, <16th	Teachers reported motor difficulties	Parent reported that the children had not been diagnosed with other disabilities				Children who received fundamental movement skills training viewed themselves as having better physical coordination, physical strength and physical fitness compared to those in the control groups.
Yu et al., 2016b	CS	100%	43 DCD 87 TD 7–10 years	MABC-2	Teacher confirmed motor difficulty					Children with DCD reported poorer physical self-concept on health, coordination, and sporting ability. Girls with DCD had a lower level of PA compared to boys with DCD or TD children.
Bonney et al., 2017a	RCT	85%	43 DCD divided into two groups Females aged 13–16 years.	MABC-2, <16th BOTMP 2nd Edition	Self-report questionnaire on perceived motor competence ADL questionnaire completed in week 1			No diagnosis of a significant medical condition known to affect motor performance was noticed nor reported.	Recruited from mainstream high school assumed no intellectual or cognitive impairment.	*Both the Task-oriented Functional Training (TFT) and Wii training groups showed significant improvement in muscular strength, motor proficiency, running and agility, predilection for physical activity and generalized self-efficacy.*
Cairney et al., 2017 Same sample as PHAST study	Long	100%	97 pDCD 1857 TD Starting age 9–10 years	Bruininks–Oseretsky Test of Motor Proficiency 1st Edition (BOT-SF).					Excluded children with known physical or learning difficulties	Cardiorespiratory fitness was lower in DCD at each time point. CRF decline for both groups over time and this was steeper in DCD. Physical activity explained a small part of the difference in CRF.
King-Dowling et al., 2018** Same sample as CATCH study	CS	88%	111 DCD 177 rDCD 301 TD Children 4–5 years	MABC-2, DCD < 5th, rDCD between 6th and 16th					Parents confirmed motor difficulties not due to another condition	There was a large main effect of DCD group on both musculoskeletal and aerobic fitness performance, children with DCD had the greatest fitness deficits. No significant group differences regarding MVPA.
Fong et al., 2018	CS	75%	52 DCD (7.5 years) 61 TD (7.2 years)	BOMPT < or equal to 42 OR MABC-2, <5th	DCD-Q				Excluded those with diagnosed disorders which would better explain the difficulties	After accounting for effects of age, gender, height, lean mass, and fat mass, the total activity diversity score remained independently associated with leg BMC in children with DCD, explaining 5.1% of the variance. PA diversity score was not associated with leg BMC.
Yu et al., 2021	CS	63%	73 DCD 99 TD 6–10 years	MABC-2, <5th	MABC-2 Checklist or the Caregiver Assessment of Movement Participation completed by teachers and/or parents				No known neurological or intellectual impairments or other medical conditions	33% of children with DCD met MVPA guidance. DCD had poorer FMS proficiency in jumping and catching and running, jumping, catching, and kicking.
**Psychological Capability**										
Cairney et al., 2005b	CS	75%	44 DCD 556 TD 9–14 years	BOTMP-SF, <10th			Excluded those with known learning disabilities/physical health problems			Generalized Self-Efficacy: Children with DCD reported lower self-efficacy to PA.
Cairney et al., 2005a	CS	88%	44 DCD 556 TD 9–14 years	BOTMP-SF, <10th			Excluded those with known learning disabilities/physical health problems			Regardless of gender, children with DCD had lower self-efficacy toward physical activity and participated in fewer organized and recreational play activities. Girls with DCD had the lowest mean scores of all children.
Poulsen et al., 2007	QE	100%	60 DCD 113 TD Mean age 11 years	MABC	Reported difficulties with tasks of daily living	No reported intellectual impairment	No reported diagnosed emotional, neurological or motor disorder			Group differences in loneliness and sports participation and social-physical participation. Relationship between MABC score and loneliness was mediated by sports participation.
Cairney et al., 2007	CS	100%	44 pDCD 546 TD 9–14 years	BOTMP-SF, <10th		Children with known learning disabilities and physical health problems were excluded				Children with probable DCD reported lower average enjoyment scores, lower perceived adequacy, higher percentage body fat and lower cardiorespiratory fitness. Negative correlation between probable DCD and enjoyment of PE.
Kane and Bell, 2009	Case series	70%	3 DCD children	BOTMP-SF	DCD-Q and leisure section of Canadian Occupational and Performance Model	Excluded if had any known neurological condition	Excluded is WISC < 70			Self-efficacy for PA is a key contributor to participation in PA.
Silman et al., 2011	CS	100%	61 pDCD 61 TD 12–13 years	MABC-2, <15th			KBIT-2 used to determine IQ			Lower perceived adequacy in DCD compared to peers. Perceived adequacy and physical activity were significant mediators. in the relationship between DCD and fitness.
Engel-Yeger et al., 2012	CS	100%	33 DCD (7.67 years) 33 TD (7.84 years)	MABC,<15th			Diagnosed by a pediatrician/neurologist			Children with DCD showed lower preference to participate in activities compared to peers.
Kwan et al., 2013 Same sample as PHAST study	CS	100%	19 pDCD 42 TD 13–14 years	MABC-2, 15th	Had MABC-2 scores across two time points with both falling below 15th, taken as indication of ADL		KBIT-2 used to determine IQ			Poorer physical activity cognitions in DCD compared to peers. Attitudes and subjective norms for PA partially mediated the relationship between DCD and PA.
Batey et al., 2014 Same sample as PHAST study	CS	88%	29 pDCD (13.3 years) 76 TD (13.2 years)	MABC-2, <15th			Excluded if <70 on Kaufman IQ test			A direct effect of DCD on PA was observed for boys, but not for girls. Neither task efficacy nor barrier efficacy influenced the relationship between DCD and PA.
Noordstar et al., 2014	CS	88%	31 DCD 31 controls 7–12 years	MABC	Reported difficulties with activities of daily living				No underlying neurological disorders were present	No difference between groups for perceived athletic competence scores, but low perceptions of athletic competence were more common in the DCD group.
Engel-Yeger et al., 2015	CS	75%	37 DCD 24 TD 6.10–9 years	MABC,<15th	All had been referred for therapy	Excluded if they had positive neurological signs/visual impairments				Children with DCD showed lower adequacy of physical activity compared to peers. Children with lower adequacy of physical activity showed lower motor performance (predicted 78% of total MABC score).
Noordstar et al., 2017	RCT	69%	Intervention group: 20 DCD Care as usual group: 11 DCD All 8 years	MABC-2	DCD-Q			Stated this met as children were between 7 and 10 yrs	Had no known neurological disorders	No effect of the different interventions on leisure PA or total PA.
Wright et al., 2019	CS	100%	60 TD 19 At risk 38 DCD 6–12 years	MABC-2, <15th rDCD, <5th DCD	Parents reported that their child had difficulty performing recreational and daily activities			Stated was all in early developmental period (not clear how determined)	Children with an intellectual disability or medical condition were excluded	Children with DCD had lower PA predilection and adequacy regarding PA, higher body fat percentage, received less logistic support. TD children had increased muscle strength compared to the DCD and at risk groups.
Zimmer et al., 2020 **	Qual	80%	6 DCD 10–12 years	MABC-2, <16th	DCD-Q				No reports of known conditions which would better explain motor difficulties	Three themes captured experiences of stress in physical education for children at risk for DCD: (1) “they hurt me” (2) “it’s hard for me” (3) “I have to.”
**Physical opportunities**										
Adams et al., 2018	QE and Qual	80%	162 Physical Therapists (survey) + plus 10 with interview data 9 DCD (interviews) 9–12 years and parents	MABC-2, <16th	Being treated or had been treated for motor difficulties by physical therapists			Considered by physical therapists treating the children	Considered by the physical therapists treating the children	*Barriers to participation included motor impairment, insufficient numbers to create a team and lack of inclusive practice.*
**Social opportunities**										
Barnett et al., 2013	Qual	70%	8 child and parent pairs All boys 13–15 years	MABC-2, <5th	MABC-checklist, <5th	BPVS score > 70	Parents reported no serious physical, sensory impairment			Majority were physically inactive but wanted to be more active. Cited poor motor skill, lack of motivation and reports of fatiguing easily, difficulty traveling to activities, negative comments from peers and teachers’ lack of understanding of DCD as barriers to increasing PA.
**Reflective motivation**										
Meek and Sugden, 1997	QE	63%	197 7-year-olds 197 11-year-olds 59 14-year-olds	TOMI-H	A checklist of behaviors associated with DCD was completed	Recruited from mainstream schools so assumed no learning, emotional or physical difficulties				No significant differences at either 7 or 11, but by 14 years of age the children with DCD had formed significantly lower attitudes than their class peers.
**Automatic motivation**										
Sit et al., 2019	RCT	69%	Intervention group: 64 DCD and 64 TD Control group: 67 DCD and 67 TD 6–10 years	MABC-2, <5th DCD and 6th–16th rDCD	MABC-2 checklist				Those with visual, neurological or intellectual impairment were excluded	Fundamental movement skills training group spent more time in MVPA and reported greater enjoyment of PA after intervention which was not the case for the control group.
Li et al., 2021 Same sample as CATCH study	CS	89%	288 rDCD 301 TD 4–5 years	MABC-2, <16th					IQ > 70 (except for one child) no other medical condition which may lead to motor impairments.	Children with rDCD reported more internalizing problems which physical activity and BMI did not mediate.

*Criteria summarized.

**Does not state which version of the diagnostic criteria they were and so have been assigned on the basis of the publication date.

CT, randomized controlled trial; CS, cross sectional; Qual, qualitative; QE, quasi-experimental; Long, longitudinal.

#### 3.1.1 Design

Studies included six randomized controlled trials (Howie et al., 2016, 2017; Yu et al., 2016a; Bonney et al., 2017a; Noordstar et al., 2017; Sit et al., 2019), four quasi-experimental studies (Meek and Sugden, 1997; Poulsen et al., 2007; Cairney et al., 2010; Green et al., 2011), 25 cross-sectional studies (Cairney et al., 2005a,b, 2007, 2019; Poulsen et al., 2008, 2011a; Baerg et al., 2011; Fong et al., 2011, 2018; Silman et al., 2011; Engel-Yeger et al., 2012, 2015; Beutum et al., 2013; Kwan et al., 2013; Batey et al., 2014; Noordstar et al., 2014; Cermak et al., 2015; Yu et al., 2016b,^2021^; King-Dowling et al., 2018, 2019; Wright et al., 2019; Brown et al., 2021; James et al., 2021; Li et al., 2021), four longitudinal studies (Cairney et al., 2006, 2017; Joshi et al., 2015; Tan et al., 2022), two qualitative studies (Barnett et al., 2013; Zimmer et al., 2020), one case series study (Kane and Bell, 2009) and one mixed-method study (Adams et al., 2018).

#### 3.1.2 Setting

Nineteen studies were conducted in Canada (Cairney et al., 2005a, b, 2006, 2007, 2010, 2017, 2019; Kane and Bell, 2009; Baerg et al., 2011; Silman et al., 2011; Kwan et al., 2013; Batey et al., 2014; Joshi et al., 2015; King-Dowling et al., 2018, 2019; Zimmer et al., 2020; Brown et al., 2021; James et al., 2021; Li et al., 2021), seven in Australia (Poulsen et al., 2007, 2008, 2011a; Beutum et al., 2013; Howie et al., 2016, 2017; Wright et al., 2019), six in Hong Kong (Fong et al., 2011, 2018; Yu et al., 2016a,b, 2021; Sit et al., 2019), three in the United Kingdom (Meek and Sugden, 1997; Green et al., 2011; Barnett et al., 2013), three in the Netherlands (Noordstar et al., 2014, 2017; Adams et al., 2018) two in Israel (Engel-Yeger et al., 2012, 2015), one in Israel and the US (Cermak et al., 2015), one in Finland (Tan et al., 2022) and one in South Africa (Bonney et al., 2017a).

#### 3.1.3 Identification of DCD

Twenty-one studies used the DSM-IV (American Psychiatric Association [APA], 1994) criteria for DCD; in line with our inclusion criteria. All administered a motor assessment (criterion A), the test component of the Movement Assessment Battery for Children second edition (MABC-2) (Henderson and Sugden, 2007) was used in 24 studies, the Bruninks Test of Motor Proficiency (BOTMP) (Bruininks and Bruininks, 1978) was used in eight studies, the test component of the Movement Assessment Battery for Children first edition (MABC) (Henderson et al., 1992) was used in six studies, a combination of the BOTMP and MABC or MABC-2 was used in three studies, the Zurich Neuromotor assessment (Largo et al., 2001) and the Test of Motor Impairment-Henderson (TOMI-H) (Stott et al., 1986) were each used in one study. A total of 12 studies described how participants met criteria B and C, and 17 described criterion D. Twenty-two studies used the DSM-5 (American Psychiatric Association [APA], 2013), in line with our inclusion criteria, all administered a motor assessment, 16 described how participants met criterion B, seven described criterion C, and 20 described criterion D. Authors typically used probable DCD (pDCD) to describe participants aged under 5 years or when all diagnostic criteria had not been assessed and at risk of DCD (rDCD) when participants fell between the 6th and 16th percentile on a standardized motor assessment. None of the studies included children under the age of 4 years old.

#### 3.1.4 Quality of the studies

Based on the study design, appropriate critical appraisal tools were used; percentage scores for methodological quality are presented in Table 2. Total percentage quality scores ranged from 62 to 100%; therefore, all included articles were of moderate to high quality. Randomized controlled trials ranged from 62 to 100%, quasi-experimental studies from 63 to 100%, cross-sectional studies from 63 to 100%, longitudinal studies from 71 to 100%, qualitative studies from 70 to 80%, the mixed-method study was 80% and case series study was 70%.

### 3.2 COM-B analysis

The results are presented within the framework of the COM-B model. A few of the included studies touched upon multiple components of the COM-B model (Yu et al., 2016a,b); these studies were aligned to a single component based on the primary focus of the study.

#### 3.2.1 Physical capability: physical strength, skill, or stamina (capacity to engage in the necessary physical processes)

Nine articles were best aligned with this component of the COM-B model (Fong et al., 2011, 2018; Joshi et al., 2015; Yu et al., 2016a,b; Bonney et al., 2017a; Cairney et al., 2017; King-Dowling et al., 2018; Li et al., 2021).

In terms of physical skill, Fong et al. (2011) used the Children’s Assessment of Participation and Enjoyment (CAPE) questionnaire (Imms, 2008) to determine whether motor ability and weight status were associated with physical activity participation diversity in children aged 6–12 years with (*N* = 81) and without (*N* = 67) DCD. Children with DCD had significantly lower CAPE total participation intensity scores than TD children. Specifically, the authors highlighted that motor ability was positively correlated with CAPE total diversity scores in children with DCD, accounting for 7.6% of the variance in CAPE total diversity scores. In other words, children with DCD who presented a higher motor skill level participated in more formal, recreational and skill-based activities. Conversely, weight status was negatively correlated with total CAPE and recreational activity diversity scores, indicating that children with higher weight status participated in fewer activities. Therefore, physical skill, motor impairment and weight status contributed to a lack of participation in physical activity in children with DCD. This is supported by Fong et al. (2018), who compared bone mineralization and activity patterns of 52 children with DCD (mean age 7.5 years) and 61 TD children (mean age 7.2 years). After accounting for age, sex, height, lean mass and fat mass, bone mineralization and activity participation were lower in children with DCD compared to TD children. The authors recommended that children with DCD should be encouraged to participate in various activities, not just physical activity, to improve bone mineralization in prepubertal years.

Taking a different approach to physical skill, Yu et al. (2016a) conducted a quasi-randomized controlled repeated measures single-blind trial to measure FMS using the Test of Gross Motor Development-2nd edition (Ulrich, 2004). They found that FMS training effectively improved both locomotor skills (jumping) and object-control skills (catching and kicking) of children aged between 8 and 10 years with DCD (*N* = 38); improvements in object-control skills (catching and throwing) were sustained for at least 6 weeks. FMS training also effectively improved the self-perceived physical competency of children with DCD in terms of physical coordination, physical strength and physical fitness immediately after the training. In a follow-up study, Yu et al. (2016b) examined differences in FMS proficiency, physical self-concept and physical activity in children aged 7–10 years with DCD (*N* = 43) and age-matched TD children (*N* = 87). They found that physical activity was correlated with FMS proficiency. Children with DCD reported significantly poorer self-concept on physical coordination and sporting ability, which was more pronounced for girls with significantly lower physical activity levels.

In a later study, Yu et al. (2021) explored differences in FMS in a large sample of children with DCD (*N* = 73) and TD children (*N* = 99) aged 8–9 years; they explored whether FMS was associated with moderate to vigorous physical activity (MVPA) and sedentary behavior. Using accelerometry to assess MVPA and five components of FMS (running, jumping, throwing, catching, kicking) from the Test of Gross Motor Development-2nd edition (Ulrich, 2004), they found that children with DCD had significantly poorer FMS proficiency in terms of specific movement patterns (jumping and catching) and outcomes (running, jumping, catching, and kicking). However, there were no significant differences in MVPA and sedentary behavior between children with DCD and TD. However, specific FMS movement patterns (running, jumping, catching) were closely related to MVPA and sedentary behavior in children, moderated by motor coordination status and sex.

In relation to physical strength and stamina, as part of the Physical Health Activity Study Team (PHAST), Joshi et al. (2015) found that children with probable DCD (pDCD; *N* = 103) had higher body mass index (BMI) and waist circumference than TD children; this difference between groups increased from baseline when the children were 9–10 years old over the 5-year study period. Boys with pDCD had a more rapid increase in BMI and waist circumference than girls with pDCD. Physical activity levels did not mediate or moderate the relationship between pDCD and measures of body composition. However, physical activity was negatively associated with measures of body composition. Likewise, using the same cohort, Cairney et al. (2017) evaluated whether physical activity levels could account for poor fitness among children with pDCD over a 5-year period. They reported that children with pDCD had poorer cardiorespiratory fitness compared to TD children; however, cardiorespiratory fitness in pDCD children at age 9 years was comparable, with a slight increase noted at age 14 years, which could not be explained by differences in self-reported physical activity at these ages.

Further evidence of differences in physical stamina and physical strength comes from King-Dowling et al. (2018), who examined differences in children at 4–5 years with DCD (*N* = 111), at risk of DCD (*N* = 177) and TD children (*N* = 301) from the Coordination and Activity Tracking in Children (CATCH) sample to determine whether vigorous physical activity (VPA) levels mediated differences in health-related fitness. They found a significant main effect of the DCD group on musculoskeletal and aerobic fitness performance; however, daily VPA was similar across groups and did not explain health-related fitness differences.

Using a gamification approach to physical strength and stamina, Bonney et al. (2017b) randomly allocated females aged 13–16 years with DCD to one of two intervention groups. The first intervention involved a 45 min Nintendo Wii session, and the second involved task-oriented functional training. Both interventions were held once weekly for 14 weeks. Blinded assessors measured outcomes at baseline and at the end of the intervention period, which included impairment-based outcomes (e.g., isometric strength), activity-based outcomes (e.g., a stair climbing test) and participation-based outcomes [e.g., Children’s Self-perceptions of Adequacy in and Predilection for Physical Activity (CSAPPA) questionnaire; Hay, 1992]. Both interventions improved muscle strength, motor proficiency, functional performance, self-efficacy and participation in activities of daily living (ADLs). In addition, although there was no statistically significant difference in aerobic stamina (running task) between pre- and post-test, significant changes were found in a predilection for physical activity and overall self-efficacy score. Improvements in participation in ADLs were also observed.

#### 3.2.2 Psychological capability: knowledge, psychological strength, skill, or stamina (capacity to engage in necessary thought processes)

Thirteen articles best aligned with the psychological capability component of the COM-B model (Cairney et al., 2005a,b; Poulsen et al., 2007; Kane and Bell, 2009; Silman et al., 2011; Engel-Yeger et al., 2012, 2015; Kwan et al., 2013; Batey et al., 2014; Noordstar et al., 2014, 2017; Wright et al., 2019; Zimmer et al., 2020).

Regarding psychological skill, Cairney et al. (2005b) quantitatively explored whether 9–14-year-old children with pDCD (*N* = 44) report lower levels of self-efficacy toward physical activity and engage in less free play and organized activities than their TD peers (*N* = 546) taking sex into account. Although girls with DCD had the lowest mean scores, all children with pDCD reported lower self-efficacy scores to participate in physical activity and lower levels of participation in free and organized play compared to children without DCD. In a follow-up study, Cairney et al. (2005a) investigated the effect of sex on the relationship between pDCD and self-reported participation in organized and recreational free-play activities. Data from 44 pDCD children and 556 TD children aged between 9 and 14 years showed that regardless of sex, children with pDCD had lower self-efficacy toward physical activity and participated in less organized and free-play activities than TD children. Again, girls with pDCD had the lowest mean scores of all children.

These findings are supported by a more recent qualitative study by Zimmer et al. (2020), who explored physical education experiences among six children at risk of DCD (10–12 years) through two semi-structured interviews with each child. To describe the stressors that children at risk of DCD experience in relation to physical education, three themes were identified using interpretative phenomenological analysis within the framework of relatedness, competence and autonomy: (a) *they hurt me*, referring to psychological and physical harm sustained from peers; (b) *it’s hard for me*, referring to difficulties in taking part in activities and (c) *I have to*, referring to perceived teacher’s demands. The authors highlight that while the stressors these children experienced interfered with fulfilling their basic psychological needs for relatedness, competence and autonomy, they primarily used coping strategies to minimize their experiences of stress.

Likewise, in a case-series study of three children aged 9–11 years with DCD, Kane and Bell (2009) evaluated a 6-week group exercise program and measured self-perceived adequacy, synonymous with psychological skill, for physical activity as one of the outcomes. Only one of the three children saw a considerable improvement in self-efficacy (pre-test: 55; post-test: 73) as measured by the Children’s Self-Perceptions of Adequacy in and Predilection for Physical Activity (Hay, 1992). While this child did not see any changes in Bruininks-Oseretsky Test of Motor Proficiency (BOTMP) (Bruininks and Bruininks, 1978) scores, the self-rated performance of their motor goals improved. One of the other children also rated their performance on their motor goals higher after intervention and had improved greatly on the BOTMP but saw little change in their self-perceived adequacy for physical activity. The third child saw little to no change in motor skills and self-efficacy. Thus, it appears that the relationship between motor performance and self-perceived psychological skills for physical activity is not the same for everyone. Kane and Bell (2009) contend that both factors likely affect participation; therefore, both should be considered important outcomes when evaluating interventions designed to increase physical activity.

In a more extensive study, Engel-Yeger et al. (2012) examined preference differences between children aged 7 years with (*N* = 33) and without (*N* = 33) DCD to participate in leisure activities, their physical activity levels as reported by their sports teacher and whether reports from their sports teacher could predict participation preferences. Significant differences were found in participation preference between groups based on the Preference for Activities of Children (PAC) (King et al., 2007) and Teacher Estimation of Activity Form (TEAF), a measure of sports performance and adequacy of physical activity (Hay, 1992). They found that TEAF scores successfully predicted children’s preference to participate in leisure activities, suggesting psychological skill is related to participation in physical activity. A more recent cross-sectional study examined the relationship between self-efficacy and motor performance in 37 children with DCD and 24 TD children (6–9 years) (Engel-Yeger et al., 2015). Children with DCD scored significantly lower on all self-efficacy scores on the Perceived Efficacy and Goal Setting (PEGS) (Missiuna et al., 2004) compared to their TD peers and sports teachers rated children with DCD significantly lower on the TEAF (Hay, 1992). Lower TEAF scores were associated with poorer motor scores on the MABC (Henderson and Sugden, 2007) and lower self-efficacy on the PEGS (Missiuna et al., 2004). The authors suggest that with failed attempts to learn motor skills due to poor motor ability, children with DCD develop lower self-perceptions and lower self-efficacy, which then creates a negative feedback loop that reduces their motivation to practice motor skills.

In a further study by Noordstar et al. (2014), they looked at the differences and relationships between perceived athletic competence and physical activity in children aged 7–12 years with (*N* = 31) and without (*N* = 31) DCD. The DCD group participated in less total physical activity than the TD children, primarily driven by less participation in unorganized physical activity in children with DCD. In relation to perceived psychological skill, no significant group differences were seen in perceived athletic competence levels. However, when the authors split both the group with DCD and the group without DCD into sub-groups with “high” or “low” perceived competence, no difference in terms of physical activity was seen between the DCD group and the TD group when their level of perceived athletic competence was low. Conversely, when perceived athletic competence was high, TD children showed greater physical activity levels than children with DCD. These findings suggest that a perception of high physical athletic competence drives physical activity in children without DCD but not in children with DCD.

Further support for poor physical ability self-concept comes from Poulsen et al. (2007), who used the Self-Description Questionnaire-I (Marsh, 1990) to examine the relationship of self-concept with patterns of physical activity in 10–13-year-old boys with (*N* = 60) and without (*N* = 113) DCD. Not surprisingly, the boys with DCD reported significantly lower physical ability self-concept than their coordinated peers. Significantly lower general and peer relations self-concept were also noted in children with DCD. Despite the small effect size, self-perceptions of peer relationships mediated low energy expenditure patterns, suggesting that the social context may have more influence on increasing physical activity than physical ability self-concept. Another recent study explored barriers and task self-efficacy toward physical activity (Batey et al., 2014). A subset of participants from the PHAST study, aged 13–14 years, were asked to complete the self-efficacy scale (Foley et al., 2008) to assess their perceived psychological stamina to complete different intensities and duration of physical activity (task efficacy) and their confidence in completing physical activity when faced with everyday barriers (barrier efficacy). An accelerometer was used to record activity for 1 week. The authors found that children with pDCD (*N* = 29) spent significantly less time in MVPA and had significantly lower task and barrier self-efficacy toward physical activity than their TD peers (*N* = 76).

Another study from the PHAST cohort was conducted by Silman et al. (2011), who examined whether perceived adequacy and physical activity mediated cardiorespiratory fitness, as measured by peak aerobic power, in children with (*N* = 61) and without (*N* = 61) pDCD at age 12–13 years. Overall, they found that children with pDCD had lower perceived adequacy toward physical activity; perceived adequacy and physical activity were significant mediators in the relationship between pDCD and peak aerobic power. In another study that utilized the PHAST cohort, Kwan et al. (2013) specifically explored the influence of physical activity cognition amongst boys aged 13–14 years with (*N* = 19) and without (*N* = 42) pDCD within the framework of the Theory of Planned Behavior (Ajzen, 1991). The authors found that boys with pDCD had poorer physical activity cognitions than TD boys. These differences were most evident in their attitude and perceived behavioral control related to being physically active, showing that the relationship between pDCD and MVPA is partially mediated by physical activity cognitions in boys with pDCD.

Similarly, Wright et al. (2019) anticipated that perceived competence, enjoyment and predilection for physical activity would be lower amongst children aged 6–12 years with DCD (*N* = 38) or at risk of DCD (*N* = 19) relative to TD children (*N* = 60). They also hypothesized that there would be a significant difference in physiological characteristics between TD children, children at risk of DCD and children with DCD and children either at risk of DCD or with DCD would have lower cardiorespiratory fitness and physical activity levels. They found that children with or at risk of DCD reported lower scores on psychological constructs that are predictive of physical activity involvement relative to TD children. Children with or at risk of DCD also had multiple physiological deficits (e.g., muscle strength) and received less parental logistic support for physical activity involvement (e.g., transportation).

Cairney et al. (2007) also explored perceived enjoyment of physical education and examined correlations between enjoyment and body fat, cardiorespiratory fitness and perceived adequacy in children with pDCD (*N* = 44) at 9–14 years. They found that children with greater perceived adequacy, lower body fat and higher cardiorespiratory fitness were more likely to enjoy physical education. They also noted that children with pDCD were more likely to be above the normal, healthy weight for their age, have poorer physical fitness and perceive themselves as less adequate (the most significant contributing factor to the enjoyment of physical education) about their physical abilities than children without pDCD.

In an intervention study, Noordstar et al. (2017) compared a motor intervention alone (*N* = 11) with a motor intervention coupled with a program to boost psychological skill (*N* = 20) in 8-year-old children with DCD. Motor control perceived self-competence and general self-esteem all improved over time; however, there were no effects of the intervention group on any of these measures. Despite these positive changes in both groups, no differences were found in physical activity levels. Therefore, it would seem, in line with the findings from Noordstar et al. (2014), that a change in perceived athletic competence does not result in a behavior change amongst 8-year-old children with DCD in this context.

#### 3.2.3 Physical opportunity: opportunities provided by the environment, such as time, location, or resource (physical opportunity provided by the environment)

Only one study considered the physical opportunity component of the COM-B model. In the only identified mixed-method study, Adams et al. (2018) explored the role of pediatric physiotherapists in promoting sports participation in children with DCD. A total of 162 physiotherapists completed a survey and 10 physiotherapists and 9 children with DCD (9–12 years) took part in interviews. Although nearly half of the physiotherapists surveyed signposted children with DCD to sports clubs, the interview data suggest that matching sports to children’s motor ability wishes and preferences facilitated participation. Identified barriers included a lack of understanding of DCD and the motor difficulties experienced by children with DCD.

#### 3.2.4 Social opportunity: opportunities as a result of social factors, such as social norms and social cues (cultural milieu that dictates the way we think about things)

There was limited literature exploring the social opportunity component of the COM-B model in the context of physical activity. One study, however, used semi-structured interviews with eight 12- to 15-year-old boys with DCD and their parents to examine barriers and facilitators to participation in physical activity (Barnett et al., 2013). Half of the children with DCD and all but one parent reported that teenagers with DCD did little physical activity. Dislike of competitive team games, lack of nearby resources, negative comments from peers and teachers, lack of motor skills and confidence, poor motivation, lack of time, fatigue and pain and lack of understanding of DCD were all constraints to participating in physical activity. In contrast, parental support and intervention activities (such as gym sessions) led to engagement and enjoyment in physical activity. The authors concluded that although teenagers with DCD disliked competitive team games, they reported many physical activities they enjoyed when social opportunities were facilitated and when they were motivated to be more physically active.

#### 3.2.5 Reflective motivation: reflective processes, such as making plans and evaluating things that have already happened (evaluations and plans)

Only one article aligned with the reflective motivation component of the COM-B model. Meek and Sugden (1997) aimed to establish whether children (aged 7–8 years; *N* = 197, 10–11 years; *N* = 197, 13–14 years; *N* = 59) with and without DCD form expectance-value combinations of attitudes prior to the completion of a novel physical activity that significantly differs from their class peers who had either previously played volleyball or not played volleyball. There were no significant between-group differences at age 7 or 11 years, but by 14 years of age, the children with DCD had formed significantly lower attitudes than their class peers. Furthermore, as age increased, attitudes decreased, suggesting that even prior to undertaking physical activity, negative attitudes existed amongst older children with DCD. Such personal barriers may interact with environmental constraints and lead to an overall lack of engagement in physical activity in teenagers with DCD.

#### 3.2.6 Automatic motivation: automatic processes, such as our desires, impulses and inhibitions (emotions and impulses arising from associative learning and/or innate dispositions)

Two articles best aligned with the automatic motivation component of the COM-B model (Sit et al., 2019; Li et al., 2021).

Li et al. (2021) examined the connections between physical activity and weight status to internalizing problems using a modified version of the environmental stress hypothesis as part of the CATCH study. They found that preschool children (4–5 years) at risk of DCD (*N* = 233) experienced more internalizing problems than their TD peers (*N* = 274), including emotion control, withdrawal from social interactions and complaints of somatic responses. Neither physical activity nor BMI mediated the relationship between children at risk of DCD and internalizing problems. It could be argued that preschool children at risk of DCD may be as physically active as their typically developing peers at this age [demonstrated in other studies described in section “3.2.1 Physical capability: physical strength, skill, or stamina (capacity to engage in the necessary physical processes)”].

In a later study, Sit et al. (2019) hypothesized that children with DCD aged 6–10 years who received FMS training would improve their motor skills proficiency and have higher physical activity levels. They perceived competence and enjoyment compared to those receiving conventional physical education. The authors concluded that children in the FMS training group improved locomotor and object control skills and engaged more in MVPA. However, there were no differences to the control group, although children with DCD did report increased enjoyment in physical activity during their leisure time, which was sustained for up to 12 months.

#### 3.2.7 Behavior: the product of perceived capability, opportunity and motivation

Fifteen articles best aligned with the behavior component of the COM-B model (Cairney et al., 2006, 2010, 2019; Poulsen et al., 2008, 2011a; Baerg et al., 2011; Green et al., 2011; Beutum et al., 2013; Batey et al., 2014; Cermak et al., 2015; Howie et al., 2016, 2017; King-Dowling et al., 2019; Brown et al., 2021; James et al., 2021; Tan et al., 2022).

King-Dowling et al. (2019), using the CATCH sample, aimed to determine if there were differences in patterns of activity levels amongst preschool children (4–5 years) with pDCD (*N* = 111), children at risk of DCD (*N* = 177) and TD children (*N* = 301). They found that preschool children with pDCD and children at risk of DCD had comparable physical activity levels to their TD peers. However, preschool children with pDCD tended to accumulate their MVPA in shorter episodes of physical activity (King-Dowling et al., 2019). This pattern is consistent with evidence from Brown et al. (2021), who also used the CATCH sample to measure the BMI of children aged 4–5 years at risk of DCD (*N* = 288) and TD age-matched children (*N* = 301). They also measured physical activity and sedentary behavior using an accelerometer whilst parents completed the Child Behavior Checklist (Achenbach, 1991). Both groups were found to engage in similar activity levels (5 h) during a 12-h awake period and movement behavior did not influence children’s mental health based on parental reports. Taken together with the findings of King-Dowling et al. (2019), these results suggest that differences in sedentary time and physical activity may develop later in childhood.

An older sample supports the conclusion that differences in physical activity behavior may appear later in childhood. Beutum et al. (2013) recruited 9 children with pDCD and 9 TD children (aged 8 years) and found that children with pDCD participated in significantly less MVPA, had higher BMI and decreased strength and cardiovascular fitness. In addition, strength, activity type and family factors correlated significantly with MVPA for children with pDCD. In a larger cross-cultural study between the United States and Israel, 53 children with DCD and 65 TD children (aged 6–11 years) were recruited to measure relationships between children’s motor coordination and their physical activity, sedentary behavior, fitness and weight status (Cermak et al., 2015). In Israel and the United States, children with DCD demonstrated significantly reduced physical activity, increased sedentary behavior, poorer fitness, and increased weight compared with TD children; no significant differences were found between the two countries. Differences in health-related fitness are also supported by the baseline data from a younger sample from the CATCH study (Cairney et al., 2019). Although no differences were observed between groups for BMI percentile or physical activity, children in the “at risk” DCD group (*N* = 287) had significantly lower aerobic and musculoskeletal fitness and larger waist circumferences compared to TD children (*N* = 301) at age 4–5 years (Cairney et al., 2019).

However, James et al. (2021) examined the effect of DCD risk amongst preschool children aged 4–5 years using the CATCH sample on MVPA levels when adjusting for ADHD symptomology. They reported that when adjusting for ADHD (particularly inattention), preschool children at risk of DCD (*N* = 288) were significantly less active than their TD peers (*N* = 301), suggesting that ADHD and DCD combined may have a negative impact on levels of physical activity in preschool-aged children. In an older PHAST sample, Baerg et al. (2011) compared physical activity using a 7-day accelerometry analysis of 12–13-year-old children with DCD (*N* = 32), children with DCD/ADHD (*N* = 30), and TD children (*N* = 48). The accelerometer was used to assess step count and activity energy expenditure of sedentary, light, moderate and vigorous levels of physical activity. The authors reported a sex and group interaction effect for average daily step counts and activity energy expenditure. Specifically, girls with DCD/ADHD had significantly more average step counts per day than TD girls. However, there was no difference between the average step count per day in girls with DCD compared to the DCD/ADHD and TD groups. There was also no significant difference between the average step count per day in boys with DCD, DCD/ADHD, or TD. The authors concluded that hyperactivity, as expressed in children with DCD/ADHD, appears to override the hypoactive behavior typically found in children with DCD. However, this finding was only found in girls and did not translate to boys with DCD/ADHD.

Cairney et al. (2006) explicitly explored whether the activity deficit between children with and without DCD widens or diminishes over time. In a cross-sectional study that administered a participation questionnaire to 44 children with DCD and 537 TD children (aged 9–14 years), they found that children with DCD participated in less structured and free play activities, but this activity deficit did not increase with age (Cairney et al., 2006). In a follow-up longitudinal study, the PHAST sample was used by Cairney et al. (2010) to recruit 111 children with pDCD and 1972 TD children at 9 years of age and followed them at ages 10 and 11 years using a participation questionnaire. The results indicate that divergence in free play activity over time occurs for females with pDCD but not for males. In another longitudinal study, Tan et al. (2022) analyzed longitudinal data to consider associations between the “at risk” status of DCD in childhood and physical activity in adulthood. Those children classified as “at risk” or “probably at risk” of DCD at 56 months were found to have a significantly lower number of steps over a 10-day period at 25 years of age compared to those children who were not at risk for DCD. Furthermore, statistical modeling indicated that DCD “risk status” increased time spent in sedentary light activity and decreased time spent in MVPA.

Other sex differences have also been noted. Using a sample from the Avon Longitudinal Study of Parents and Children (ALSPAC) sample, Green et al. (2011) explored whether children with pDCD (*N* = 193) had an increased risk of reduced MVPA compared to TD children (*N* = 4,138) at two time points (t1: 7–8 years; t2: 12–13 years) using accelerometry for 7 days. Boys with pDCD were less physically active than boys without pDCD at ages 7 and 12 years. There were no differences in levels of MVPA in girls with and without pDCD, which the authors suggest may reflect a generally low level of MVPA across the entire sample. Additionally, Poulsen (2008) asked parents of 60 boys aged 10–13 years with DCD and 113 boys without DCD to complete a 7-day leisure time diary and record the intensity, duration, content and social/physical environment of leisure time activities. A total daily score for low-intensity activities (LPA) and MVPA and the total metabolic (MET) levels were computed for 1 week’s activities. Boys with DCD spent significantly less time engaged in MVPA compared to boys without DCD but spent significantly more time in LPA. This pattern of leisure physical activity contributed to significantly lower energy expenditure in boys with DCD compared to their peers. Interestingly, the highest percentage of out-of-school time for both groups was devoted to sedentary, unstructured pursuits (e.g., television, electronic media). Boys with DCD had significantly lower participation in structured (e.g., team sports) and unstructured (e.g., street ball, running games) social physical activities; no significant differences were noted between groups for physical non-social activities (e.g., individual sports). In a follow-up study, Poulsen et al. (2011b) explored the differences in the number and context of leisure-time personal projects reported in boys with and without DCD. Group-matched 10–12-year-old boys with (*N* = 60) and without DCD (*N* = 113) completed the Personal Project Analysis for Children (Christiansen, 2000). Boys with DCD identified significantly fewer MVPA, team sports, popular sports and structured physical activity personal projects than boys without DCD. Boys with DCD accounted for 81.4% of participants involved in no team sports and 74.4% of participants who participated in two or fewer activities involving MVPA. Furthermore, the majority of physical activity reported by participants with DCD were completed individually or in the home environment.

In terms of exploring whether interventions can improve physical activity levels in children with DCD, Howie et al. (2016) recruited 21 children with DCD or at risk of DCD (aged 10–12 years) to take part in a crossover active video game (AVG) intervention. The intervention (AVG, no AVG) periods were 16 weeks for 20 min a day, 4–5 days per week. Accelerometers at baseline and following each intervention period measured minutes of sedentary, light, moderate and vigorous durations alongside self-reported activity types. The authors found that the AVG intervention did not improve physical activity or sedentary time. In a follow-up study to determine barriers to interventions, Howie et al. (2017) considered why the AVG intervention did not increase physical activity in the same sample. Although some participants (*N* = 5) significantly increased their physical activity following the intervention, this was not the case for all participants. In addition, there were no relationships between engagement with the AVG in terms of playing time and changes in physical activity, suggesting that levels of engagement did not explain these individual differences. Therefore, the exact barriers to AVG interventions remain unclear.

## 4 Discussion

The evidence relating to physical capability was of moderate to high quality (63–100%) and suggests that children with DCD have poorer motor skills, lower bone mineralization and participate in less varied formal, recreational and skill-based activities (Fong et al., 2011, 2018). Children with DCD also have higher BMI and waist circumference, which is especially the case for boys (Joshi et al., 2015). Physical activity levels do not seem to account for these differences (Joshi et al., 2015), nor does physical activity mediate the poorer cardiorespiratory fitness seen in children with DCD (Cairney et al., 2017) and VPA does not explain differences in health-related fitness (King-Dowling et al., 2018), although it is possible that lower levels of physical activity in DCD may be a consequence of these differences. FMS differences have also been found in children with DCD (Yu et al., 2021), with FMS proficiency correlated with physical activity (Yu et al., 2016b). In addition, there is evidence that FMS proficiency can be improved with FMS training (Yu et al., 2016a). Both Nintendo Wii and task-oriented functional interventions appear to improve muscle strength, motor proficiency, functional performance, self-efficacy and participation in ADLs (Bonney et al., 2017a), at least in the short term. In the context of physical capability, the evidence suggests that children with DCD have poorer physical skills and physical strength, resulting in poorer physical stamina.

Overall, the evidence relating to the psychological capability, which was of moderate to high quality (69–100%), suggests that children with DCD have lower levels of self-efficacy and perceived athletic competence toward physical activity, with the lowest self-efficacy reported amongst girls (Cairney et al., 2005a,b; Poulsen et al., 2007; Silman et al., 2011; Kwan et al., 2013; Batey et al., 2014; Wright et al., 2019), despite fitness differences being found in boys with DCD. Qualitative research also suggests that the stressors experienced by children with DCD around compulsory physical education are often managed using coping strategies (Zimmer et al., 2020), which are important self-management approaches given that physical activity interventions may not improve motor skills or self-efficacy (Kane and Bell, 2009). As a result, children with DCD may instead develop lower self-perceptions, lower self-efficacy and perceive themselves as less adequate in their physical abilities than children without DCD (Cairney et al., 2007), creating a negative feedback loop that reduces their motivation to practice motor skills and participate in physical activity (Engel-Yeger et al., 2012, 2015). Interestingly, perceptions of high physical athletic competence may drive physical activity in children without DCD but not in children with DCD (Noordstar et al., 2014, 2017) (see Dreiskämper et al., 2022 for an extensive discussion of this in TD children).

One article of high quality (80%) aligned with the physical opportunity component of the COM-B and identified that signposting children with DCD to sports clubs required consideration of children’s motor skills, wishes and preferences with a lack of understanding identified as a barrier to participation (Adams et al., 2018). Social opportunity, lack of motor skills and confidence, poor motivation, lack of time and fatigue and pain are all reported barriers to participation in physical activity (Barnett et al., 2013) based on one high-quality study (70%). The evidence relating to reflective and automatic motivation, which was of moderate to high quality (63–100%), suggests that young people with DCD have negative attitudes toward physical activity (Meek and Sugden, 1997). Even preschool children at risk of DCD have greater internalizing problems than their typically developing peers (Li et al., 2021). However, one study found that following an FMS intervention, children with DCD reported increased enjoyment in physical activity during their leisure time, which was sustained for up to 12 months (Sit et al., 2019).

Taken together, the evidence aligning with the behavior component of the COM-B, which was of moderate to high quality (62–100%), suggests that levels of physical activity appear unaffected in pre-school children with DCD (Cairney et al., 2019; King-Dowling et al., 2019; Brown et al., 2021) unless there is co-occurring ADHD (James et al., 2021). Older children with DCD (>6 years) have lower step counts (Tan et al., 2022), lower levels of LPA and MVPA (Poulsen et al., 2008; Beutum et al., 2013; Cermak et al., 2015), higher BMI, decreased strength (Beutum et al., 2013), poorer fitness (Cermak et al., 2015) and participate in less structured and free play activities which do not change with age (Cairney et al., 2006), although divergence in free play activities was found for females with pDCD over time (Cairney et al., 2010). Other sex differences have also been noted whereby, compared to girls, boys with pDCD are generally less physically fit (Baerg et al., 2011; Green et al., 2011) and engage in less physical activity (Batey et al., 2014). In terms of improving physical activity levels, there is currently insufficient evidence to support the implementation of home-based AVG interventions for children aged 10–12 years with DCD (Howie et al., 2016, 2017).

### 4.1 Strengths and limitations

This is the first systematic review that has considered physical activity amongst children with DCD in the context of a well-established behavior change model, the COM-B (Michie et al., 2011). The conduct of this review was supported by a multi-disciplinary team specializing in DCD research. The review followed the JBI methodology, which is well known for the conduct of rigorous evidence synthesis to promote and implement evidence-based decisions. Using JBI critical appraisal tools allowed for a detailed and nuanced assessment of different study designs.

However, the strict adherence to the eligibility criteria, specifically the need for authors to have explicitly stated how two or more of the DCD diagnostic criteria had been met, may have resulted in some relevant papers not being included. Furthermore, multiple studies drew on the same sample from the PHAST study between 2010 and 2017 (Cairney et al., 2010, 2017; Baerg et al., 2011; Kwan et al., 2013; Batey et al., 2014; Joshi et al., 2015) and the CATCH study between 2018 and 2021 (King-Dowling et al., 2018, 2019; Cairney et al., 2019; Brown et al., 2021; James et al., 2021; Li et al., 2021). These samples may not capture the demographic heterogeneity of the wider DCD population.

### 4.2 Future research

Based on the COM-B model of behavior change (Michie et al., 2011), future research could consider the reflective motivation and physical and social opportunities for children with DCD to engage in physical activity, an area generally neglected to date. Furthermore, based on the reviewed literature, there appear to be inconsistencies in implementing the diagnostic criteria for DCD in research. There were limited examples of all diagnostic criteria being considered. Therefore, future research should ensure the careful description of all criteria before grouping samples as DCD, rDCD, pDCD and TD. This will enable a more precise picture to emerge and opportunities for meta-analysis. In addition, only one identified study considered physical activity in adults with DCD (Tan et al., 2022). There is, therefore, a gap in understanding the capability, opportunity, motivation and behavior of adults with DCD in the context of physical activity.

### 4.3 Practical implications and recommendations

There is some evidence suggesting that FMS training (Yu et al., 2016a), Nintendo Wii interventions, and task-oriented functional interventions (Bonney et al., 2017a) may improve physical capability and that this, in turn, may improve participation in physical activity. However, recommendations for future interventions can be derived from the data obtained in this systematic review using the Behavior Change Wheel (BCW). The COM-B model forms the hub of the BCW, a systematic behavioral science tool for developing and characterizing interventions for health behavior change (Michie et al., 2011). The BCW is a synthesis of 19 behavior change models described in the literature and was developed because other existing models do not account for the full range of possible interventions for systematic health promotion intervention planning (Atkins and Michie, 2015). The BCW sits around the COM-B model and provides nine intervention functions. These are categories through which behavior can be changed: (i) training (e.g., feedback on behavior; self-monitoring of behavior, instruction on how to perform a behavior); (ii) enablement (e.g., social support, goal setting, action planning, coping planning, self-monitoring of behavior); (iii) coercion (e.g., feedback on behavior, social comparison); (iv) education (e.g., information about health consequences, feedback on behavior; prompts, cues; self-monitoring of behavior); (v) environmental change; (vi) role models; (vii) persuasion (e.g., information about health consequences, feedback on behavior); (viii) incentive (e.g., feedback on behavior; self-monitoring of behavior); and (ix) restrictions.

Based on this framework, for example, interventions to enhance the perceived psychological capability of children with DCD could include training people involved in providing physical activity opportunities to enable greater differentiation. Likewise, to enhance the social opportunity for physical activity, interventions could consider facilitating family or matched peer-based physical activities as part of daily routines. Interventions might include restructuring the environment to facilitate failure-free physical activity opportunities, preferably from a young age, to enhance reflective and automatic motivation.

## 5 Conclusion

Although preschool-aged children with DCD may engage in similar levels of physical activity behavior, differences emerge from 6 years of age; this age may align with greater expectations but also increased self-evaluation. Due to the nature of DCD, children’s reduced physical capability results in less participation in varied formal, recreational and skill-based activities, which limits their opportunity to enhance their physical capability. This may impact psychological capability, whereby children with DCD develop lower self-perceptions and lower self-efficacy, which feeds into this negative feedback loop that reduces their motivation to participate in physical activity. Barriers relating to physical and social opportunities to participate in physical activity have been identified that may result in negative attitudes and poor reflective and automatic motivation toward physical activity; however, there is some evidence that interventions, for example, using a Nintendo Wii or active video games, can enhance enjoyment, at least in the short-term. In the context of physical education, there is some indication that some children with DCD adopt coping strategies to minimize the psychological impact of compulsory participation in physical activity; however, the sustainability of adopting top-down cognitive strategies needs further investigation.

## Author contributions

CP: conceptualization, data curation, formal analysis, investigation, methodology, project administration, resources, validation, visualization, writing – original draft, Writing – review and editing. KW: conceptualization, data curation, formal analysis, investigation, methodology, validation, visualization, writing – review and editing. NS: conceptualization, formal analysis, methodology, validation, writing – review and editing. JZ: conceptualization, formal analysis, methodology, validation, writing – review and editing. VR: formal analysis, validation, writing – review and editing.
