# Supplementary material for: Understanding factors that influence physical activity behavior in people with developmental coordination disorder (DCD): a mixed-methods convergent integrated systematic review
Source: Front Hum Neurosci. 2023 Dec 13;17:1274510. doi: 10.3389/fnhum.2023.1274510 (PMC10751368; doi:10.3389/fnhum.2023.1274510)
Supplement: Supplementary file 1 [file Data_Sheet_1.docx]

Supplementary Material

**Supplementary information S1**

**Data Extraction Form**

**Accession date:**

**Reviewer (abbreviation):**

**Title**:

| Author | Journal | Publication year | Design | Sample characteristics | Methodology | Summary outcomes |
| --- | --- | --- | --- | --- | --- | --- |
|  |  |  |  |  |  |  |

**Inclusion criteria:**

Qualitative, quantitative or mixed-method

Peer reviewed

Two out of four DSM or ICD criteria for DCD

Primary focus DCD

Standardized motor assessment included

For intervention studies baseline data reported

For intervention studies comparator either did not receive PA intervention or were TD

**Exclusion criteria:**

Systematic review

Study or review protocol

Commentary

Editorial

Grey literature

Conference abstract/poster

Motor difficulties could be due to lack of opportunity

Visual impairment or other condition reported that could explain motor difficulties

Other: ________________________
